# Dr. Alfred C. West

**Published:** 1917-04

**Authors:** 


**Dr. Alfred C. West** (not listed in Polk nor State Directory, of Hornell, died in the Willard State Hospital Feb. 24, aged 100 years, 5 months and 12 days.

